# Fabrication of Magnetic Nanofibers by Needleless Electrospinning from a Self-Assembling Polymer Ferrofluid Cone Array

**DOI:** 10.3390/nano7090277

**Published:** 2017-09-17

**Authors:** Weilong Huang, Bin Liu, Zhipeng Chen, Hongjian Wang, Lei Ren, Jiaming Jiao, Lin Zhuang, Jie Luo, Lelun Jiang

**Affiliations:** 1 School of Engineering, Guangdong Provincial Key Laboratory of Sensor Technology and Biomedical Instrument, Sun Yat-Sen University, Guangzhou 510006, China; weilong_huang@foxmail.com (W.H.); liub78@mail.sysu.edu.cn (B.L.); asm0844@163.com (Z.C.); Wanghjsysu@foxmail.com (H.W.); rlei@mail2.sysu.edu.cn (L.R.); jiaojm@outlook.com (J.J.); jluo_bme@foxmail.com (J.L.); 2 School of Physics, Sun Yat-Sen University, Guangzhou 510275, China; stszhl@mail.sysu.edu.cn

**Keywords:** ferrofluid, self-assembling, needleless electrospinning, magnetic nanofibers

## Abstract

Magnetic nanofiber has been widely applied in biomedical fields due to its distinctive size, morphology, and properties. We proposed a novel needleless electrospinning method to prepare magnetic nanofibers from the self-assembling “Taylor cones” of poly(vinyl pyrrolidone) (PVP)/Fe_3_O_4_ ferrofluid (PFF) under the coincident magnetic and electric fields. The results demonstrated that a static PFF Rosensweig instability with a conical protrusion could be obtained under the magnetic field. The tip of the protrusion emitted an electrospinning jet under the coincident magnetic and electric fields. The needleless electrospinning showed a similar process phenomenon in comparison with conventional electrospinning. The prepared nanofibers were composed of Fe_3_O_4_ particles and PVP polymer. The Fe_3_O_4_ particles aggregated inside and on the surface of the nanofibers. The nanofibers prepared by needleless electrospinning exhibited similar morphology compared with the conventionally electrospun nanofibers. The nanofibers also exhibited good ferromagnetic and magnetic field responsive properties.

## 1. Introduction

Polymer magnetic composites are an exciting new class of multifunctional materials, where magnetic particles are incorporated into solid polymer matrices [1]. Polymer magnetic nanofiber is one of the most important one-dimensional (1D) magnetic materials due to its distinctive size, morphology, and unique magnetic properties [1]. The magnetic nanofibers are sensitive to change in external magnetic or electric field and can be applied in various fields, such as hyperthermia therapy [2], oil/water separation [3], targeting drug delivery [4], tissue engineering [5], microwave absorption [6], electronic and photoelectronic devices [7], and electromagnetic interference shielding [8], and so on. Therefore, magnetic nanofiber is worth synthesizing due to its broad applications.

Many synthetic approaches have been explored to prepare 1D magnetic nanostructures, including template-assisted growth [9], self-assembly [10], hydrothermal or solvothermal synthesis [11], nanolithography [12], metallization of DNA [13], electrospinning [14], and so on. Electrospinning has been demonstrated to be a versatile and feasible method to fabricate multifunctional composite nanofibers through incorporating various functionalities into the polymer solution with different nanofillers [15]. In conventional electrospinning, a high voltage is applied to a droplet of polymer solution to form the “Taylor cone”. As the applied electric field is sufficiently high, the electrostatic force can overcome the surface tension of the polymer solution and exhaust a charged jet from the tip of the “Taylor cone”. The charged jet then undergoes a stretching and whipping process, finally depositing on the counter-electrode in the formation of continuous nanofibers [16]. At present, the droplet is typically suspended at the edge of a needle attached to a vessel filled with polymer solution. A single jet is issued from a single needle, so the output of nanofibers is limited. Therefore, multi-needle electrospinning is employed to achieve a high production rate [17]. Multi-needle electrospinning is technologically inconvenient due to its complexity and high probability of clogging [18]. Needleless electrospinning methods can well solve the mass production and needle clogging problems [19]. Researchers have reported various needleless electrospinning techniques, such as porous spinnerets [20], wire spinnerets [21,22], rotary cylinder/cone/disk/convex slot spinnerets [23,24,25,26], bubble spinnerets [27], tip electrospinning of the liquid surface [28], and so on. Most of the needleless electrospinning techniques require extra power, such as the rotation of a roller driven by a motor, to form the “Taylor cones” for the electrospinning. The magnetic field can be used to form the “Taylor cones” of a ferrofluid. The Rosensweig instability of a magnetized ferrofluid has an electric analogue in the “Taylor cone” instability of an electrified fluid [29]. Yarin et al. [30] reported a magnetic-field-assisted needleless electrospinning method, which used a magnetic field to induce the formation of spikes on the solution surface and then initiated an electrospinning process. It is a two-layer system and an immiscible electrospinning solution is necessary to electrospin the nanofibers, which limits its further application. Magnetic nanofibers fabricated by this method have not been reported. 

In this study, we propose a novel fabrication method of needleless electrospinning of the magnetic nanofibers from the self-assembling “Taylor cones” of a ferrofluid under simultaneous magnetic and electric fields. The “Taylor cones” of the ferrofluid were directly self-assembled under the permanent magnets without any extra power. The needleless electrospinning setup is extremely simple and cost-effective. The multiple parallel electromagnetostatic jets can be realized through the application of simultaneous magnetic and electric fields to the electrically-conducting ferrofluid with no needles [31]. Multiple parallel jets of a ferrofluid, directly used to electrospin the magnetic nanofibers, have not been investigated yet. Furthermore, the throughput of magnetic nanofibers can be easily controlled through tuning the number of “Taylor cones”. In the next work, we will prepare a PVP/Fe_3_O_4_ ferrofluid (short for PFF) for the needleless electrospinning of the nanofibers. The Rosensweig instability of ferrofluid and needleless electrospinning process under the simultaneous magnetic and electric fields also will be observed. The morphology and magnetic properties of PVP/Fe_3_O_4_ nanofibers will be analyzed. We hope the magnetic nanofibers prepared in this work will be applied in biomedical fields, such as hyperthermia therapy and tissue engineering.

## 2. Results and Discussion

### 2.1. Static Self-Assembling of PFF Droplet under Coincident Electric and Magnetic Fields

**Figure 1:**
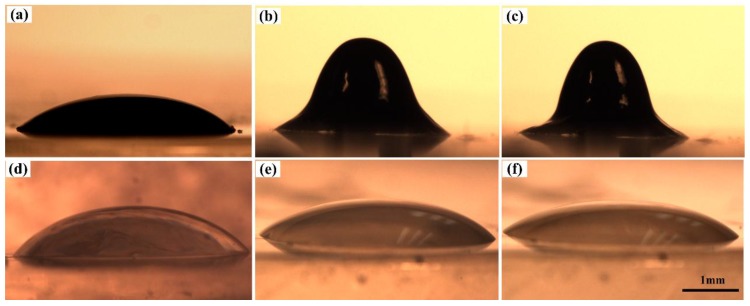
PVP/Fe_3_O_4_ ferrofluid (PFF) droplet morphology (**a**) in air, (**b**) under the magnetic field, and (**c**) under the coincident magnetic and electric fields. Pure PVP droplet morphology (**d**) in air, (**e**) under the magnetic field, and (**f**) under the coincident magnetic and electric fields. The applied external magnetic field and electric voltage were about 35 mT and 5 kV, respectively. All the subfigures share the same scale.

The interaction between the adjacent droplets can be ignored due to their size and distribution [32], so we take one droplet as the research object. Figure 1a–c present the formation behavior of a 2 μL PFF droplet through the application of magnetic and electric fields. Both the 40-mm-diameter grade-N40 large permanent magnet and the 1.1-mm-thickness, 1.9-mm-diameter grade-N40 small permanent magnets were employed to generate an external magnetic field. The applied external magnetic field intensity was 35 mT and the electric voltage was 5 kV. The PFF droplet lies quiescently and spreads out on the top surface of polydimethylsiloxane (PDMS) in the absence of applied fields, as shown in Figure 1a. Its contact angle is about 39°, which indicates the PFF wettability on the surface of PDMS, though PDMS has a relatively low surface tension [33]. The spreading droplet is drawn into an identical pointed cone geometry when the magnetic field is applied on the PFF droplet, as shown in Figure 1b, which is the Rosensweig instability of the PFF droplet by the static self-assembling under the magnetic field. The identical pointed geometry is slightly stretched in the normal direction of the droplet surface as the electric field (below the order of 108 V/m) is applied, as shown in Figure 1c. The same volume pure PVP droplet was employed to repeat the above experiment. The PVP droplet always spreads out on the surface of the PDMS block and its geometry varies little with or without application of magnetic and electric fields, as shown in Figure 1d–f. The contact angle of the PVP droplet is also about 39°. This indicates that the magnetic and electric fields have little influence on the pure PVP droplet in comparison with the PFF droplet.

Self-assembly is a process in which interacting bodies are autonomously driven into ordered structures by free-energy gradients [32]. The static self-assembling behavior of PFF a droplet can be analyzed on the potential energy of applied fields and their interaction with the fluid volume. As the net energy of the system reaches a minimum value, the equilibrium shape of fluid appears [32]. In the absence of applied field, the PFF droplet lies on the PDMS due to the simultaneous minimization of the gravitational and surface tension potential energy, as shown in Figure 1a. As a magnetic field is applied to the PFF droplet, a curved droplet surface (Rosensweig surface) is stretched and protrudes in the vertical direction [34], as shown in Figure 1b. This increases the gravitational and surface tension potential energy over a flat droplet, but simultaneously decreases the magnetic potential energy due to the PFF droplet features aligned with the magnetic field [29]. The magnetic potential energy increases with the extension of the PFF droplet along the magnetic field where the magnetic field gradient is great, as shown in Figure 7b. The iron pillar serving as an electrode can generate a high magnetic field gradient at its top ends, resulting in the formation the cone array of PFF. A greater magnetic field gradient can induce and transfer a larger magnetic force on the PFF droplet, sharpening the PFF droplet along the magnetic field line. A new balance is reached as the sum of gravity, surface tension, and magnetic force is zero, or the net energy of the system is minimized. The magnetostatic Rosensweig surface created in the PFF serves the same role as the electrified capillary in conventional electrospinning. With the application of coincident magnetic and electric fields on a PFF droplet, the curved PFF droplet surface is stretched and grows slightly taller by the electrostatic force, as shown in Figure 1c. The applied electric voltage here is, relatively, too low to form the “Taylor Cone”. The magnetic force acts together with the electrostatic traction to deform the meniscus. If the electric voltage is increased further, the meniscus can be further stressed by the electric field and results in the eventual spray from the cone tip [29].

### 2.2. Needleless Electrospinning Process

**Figure 2:**
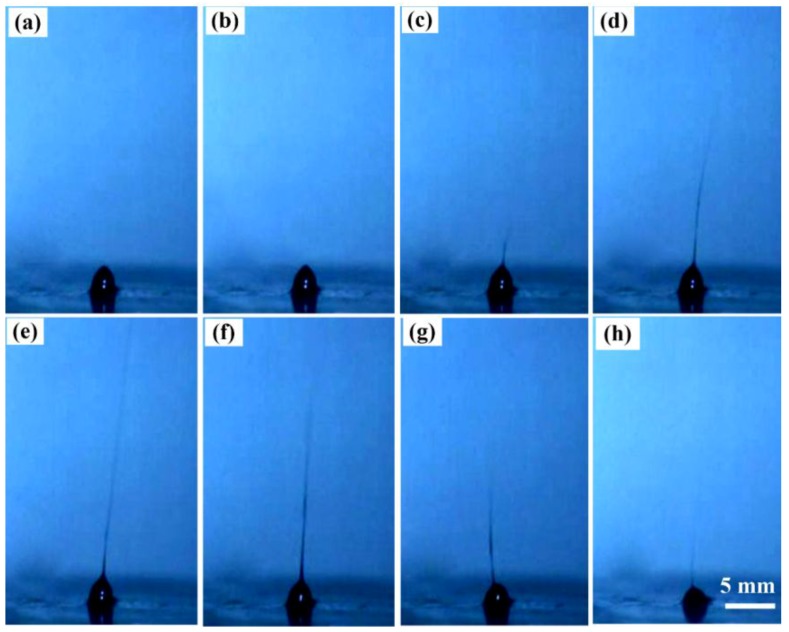
Needleless electrospinning process captured by a high-speed camera. (**a**) Self-assembling of ferrofluid, (**b**) formation of Taylor Cone, (**c**) onset of jet emission, (**d**–**f**) electrospinning process, (**g**,**h**) diminution of needless electrospinning. All the subfigures share the same scale.

The needleless electrospinning process of one PFF droplet was captured by a high-speed camera at 2000 fps and analyzed frame by frame, as shown in Figure 2. The curved peak is self-assembled under the magnetic field, as shown in Figure 2a, which is identical with that in Figure 1b. The peak is electrified and large quantities of electric charge are accumulated at the apex as the high voltage is applied. The peak becomes sharper and a “Taylor cone” forms due to the electrostatic force, as shown in Figure 2b. Once the critical threshold voltage is reached, the curved peak of the droplet abruptly becomes a sharp point, triggering the onset of jet emission from the “Taylor cone”, as shown in Figure 2c. Electrospinning occurs as the electrostatic surface traction exceeds the PFF droplet surface tension. The jet is stretched, elongated and whipped by electrostatic repulsion in the electric field, as shown in Figure 2d–f. The jet dries with the solvent evaporation in flight, becoming finer and finer, and ultimately deposits the magnetic nanofibers on the grounded collector. The jet tapers off as the applied high voltage on PVP/Fe_3_O_4_ ferrofluid is turned down, resulting in diminution of needless electrospinning, as shown in Figure 2g,h. This process is similar with the conventional electrospinning process. Conventional electrospinning is an exclusively electrostatic process, while the needleless electrospinning is a magnetoelectrostatic process. The multiple parallel cone array of PFF droplets can be simultaneously self-assembled to electrospin nanofibers under the coincident electric and magnetic fields.

### 2.3. Morphology of Nanofibers

**Figure 3:**
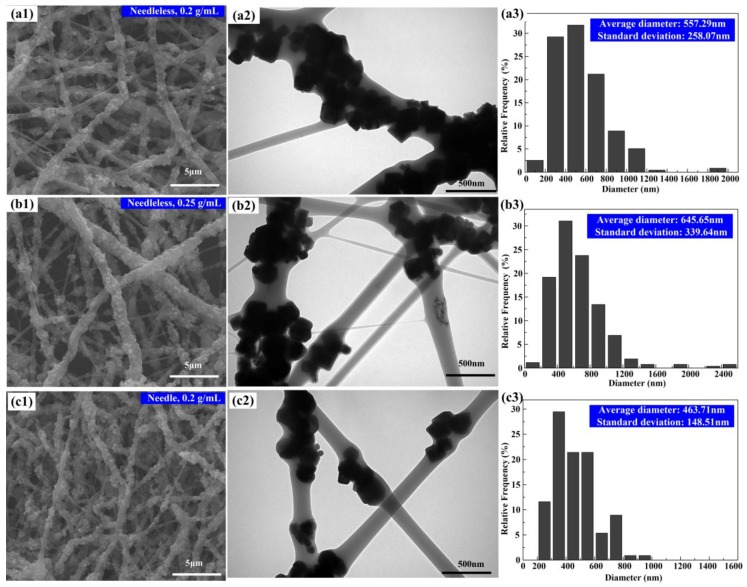
SEM images, TEM images, and the respective diameter histogram, of PVP/Fe_3_O_4_ nanofibers. Nanofibers fabricated by multiple needleless electrospinning with Fe_3_O_4_ concentration of (**a**) 0.2 g/mL and (**b**) 0.25 g/mL. (**c**) Nanofibers fabricated by conventional electrospinning with a Fe_3_O_4_ concentration of 0.2 g/mL.

The SEM images of PVP/Fe_3_O_4_ nanofibers are shown in Figure 3(a1,b1,c1). As observed, the collected nanofibers all display a random orientation due to the bending instability associated with the spinning jet [35]. The morphology of nanofibers prepared by the needleless electrospinning is similar to those by the conventional electrospinning. The surfaces of nanofibers are all rough due to the Fe_3_O_4_ particles wrapped on their surface. The inner microstructures of the nanofibers were further investigated by TEM, as shown in Figure 3(a2,b2,c2). As observed, the Fe_3_O_4_ particles are dispersed both in and on the surface of the nanofibers. Most particles are wrapped in the nanofibers, while some sections of nanofibers contain pure PVP without any Fe_3_O_4_ particles inside. The aggregation of the Fe_3_O_4_ nanoparticles in the nanofibers may be attributed to the following possible reasons: (1) the Fe_3_O_4_ nanoparticles are distributed uniformly in PFF due to the different densities between the PVP and nanoparticles. (2) The Fe_3_O_4_ nanoparticles tend to agglomerate each other to reduce its own energy in the PFF due to their high surface-to-volume ratio by electrostatic attraction. (3) The external magnetic field during the needleless electrospinning may further exacerbate the aggregation of Fe_3_O_4_ particles in the PFF due to the magnetic attraction. The cumulative frequency distribution of the electrospun nanofiber diameters is shown in Figure 3a3,b3,c3. The average diameters of nanofibers are 557 ± 258 nm, 645 ± 340 nm, and 464 ± 149 nm for the Fe_3_O_4_ particle concentrations of 0.2 g/mL needleless, 0.25 g/mL needleless, and 0.2 g/mL needle electrospinning, respectively. The average diameter of nanofibers by needleless electrospinning increases with the Fe_3_O_4_ particle concentration of PFF. It may be explained by the viscosity of PFF increasing with the Fe_3_O_4_ particle concentration of PFF. The standard deviation of needleless electrospinning nanofiber diameter is larger in comparison with conventional electrospinning. It may indicate that the needle electrospinning process is more unstable. Therefore, the needleless electrospinning can prepare magnetic nanofibers with multiple parallel jets under the coincident electric and magnetic fields.

### 2.4. TGA Analysis

**Figure 4:**
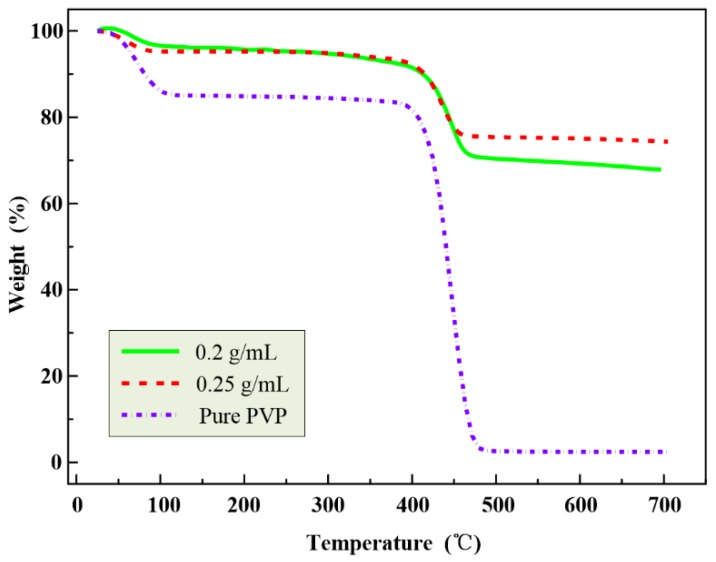
TGA curves of PVP/Fe_3_O_4_ and pure PVP nanofibers.

The thermal degradations of PVP/Fe_3_O_4_ and neat PVP nanofibers were evaluated by measuring weight loss vs. temperature of the nanofibers. TGA curves of PVP/Fe_3_O_4_ and neat PVP nanofibers are shown in Figure 4. A minor weight loss at approximately 100 °C is showed in the TGA curves. It is related to the loss of moisture absorbed by the nanofibers and the solvent trapped in the fibers [36]. A major weight loss is observed from 410 °C to 480 °C in the TGA curves due to the combustion of organic PVP matrix. No further weight loss is observed up to 700 °C, which indicates that the polymer has been completely removed. The substance left is Fe_3_O_4_ particles. The degradation temperature of Fe_3_O_4_ is beyond 1000 °C. The weights of Fe_3_O_4_ particles left are 75% and 77% for the Fe_3_O_4_ concentrations of PFF at 0.2 g/mL and 0.25 g/mL, respectively. The concentration of PVP/ethanol solution is 7 wt%. The theoretically weights of Fe_3_O_4_ in the nanofibers are 74% at 0.2 g/mL and 78% at 0.25 g/mL. Therefore, the experimental TGA values agree with the theoretical ones.

### 2.5. Fourier Transform Infrared Spectroscopy (FT-IR) Analysis

**Figure 5:**
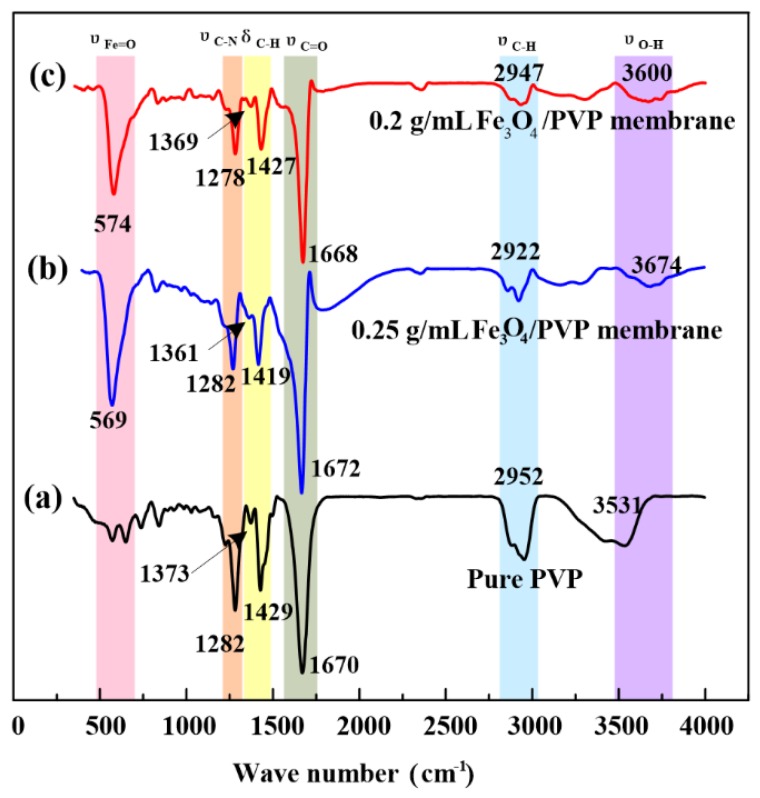
FT-IR spectra of (**a**) neat PVP nanofibers, (**b**) PVP/Fe_3_O_4_ nanofibers at 0.25 g/mL Fe_3_O_4_ particle concentration, and (**c**) PVP/Fe_3_O_4_ nanofibers at 0.2 g/mL Fe_3_O_4_ particle concentration.

Figure 5 shows the FT-IR spectra of PVP/Fe_3_O_4_ nanofibers prepared by needleless electrospinning and neat PVP nanofibers by conventional electrospinning. The main absorption peaks of neat PVP shown in Figure 5a at 3531 cm^−1^, 2952 cm^−1^, 1429 cm^−1^, 1670 cm^−1^, and 1282cm^−1^ are assigned to the O–H stretching, C–H stretching, C–H bending, C=O stretching vibration, and the tertiary amine and C–N vibration, respectively. These absorption peaks observed confirm the existence of PVP groups which are also presented in the FT-IR spectra of PVP/Fe_3_O_4_ nanofibers, as shown in Figure 5b,c. However, an extra band of the metal oxide Fe–O at 570 cm^−1^ is observed in the FT-IR spectra of PVP/Fe_3_O_4_ nanofibers. The absorption peak of Fe–O was at around 551 cm^−1^, according to the previous research [37]. This slight shift of Fe–O absorption peak may be attributed to the interaction between Fe_3_O_4_ nanoparticles and nanofibers [37]. The FT-IR spectrum of PVP/Fe_3_O_4_ nanofibers at the Fe_3_O_4_ concentration of 0.25 g/mL shows a similar profile with that of PVP/Fe_3_O_4_ nanofibers at 0.2 g/mL.

### 2.6. Magnetic Properties Analysis

**Figure 6:**
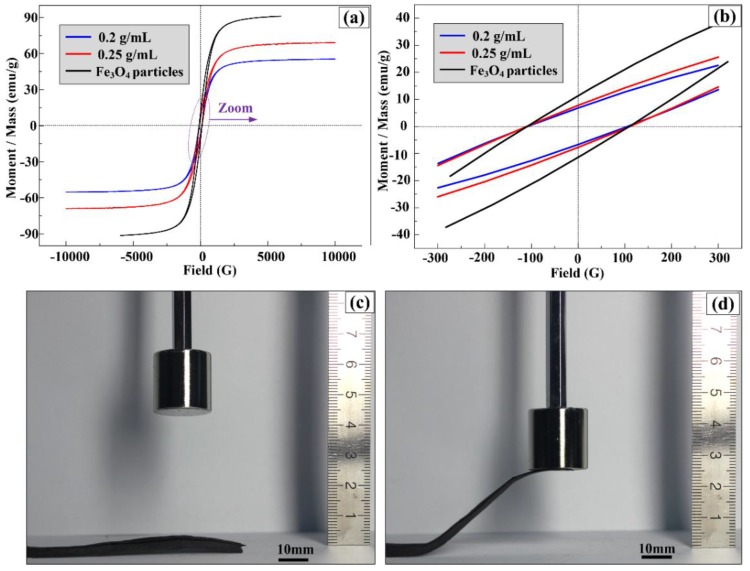
(**a**) Magnetic hysteresis loops of Fe_3_O_4_ particles and PVP/Fe_3_O_4_ membranes, (**b**) detailed view of the magnetic hysteresis loops at the origin, and (**c**,**d**) the magnetic field responsive behavior of PVP/Fe_3_O_4_ membrane.

Magnetic hysteresis loops of PVP/Fe_3_O_4_ membranes prepared by needleless electrospinning and Fe_3_O_4_ particles were obtained by VSM at room temperature, as shown in Figure 6a,b. Hysteresis loops misalign for both Fe_3_O_4_ particles and PVP/Fe_3_O_4_ membranes, which means they are ferromagnetic. The specific saturation magnetization (*M_s_*) values for pure Fe_3_O_4_ particles, 0.25 g/mL and 0.2 g/mL PVP/Fe_3_O_4_ membranes are 91 emu/g, 70 emu/g, and 55 emu/g, respectively. The decrease in saturation magnetization value of Fe_3_O_4_/PVP composite nanofibers in comparison with Fe_3_O_4_ nanoparticles can be explained as: firstly, the existence of nonmagnetic PVP components encapsulate Fe_3_O_4_ nanoparticles, which affects the magnetization expression of Fe_3_O_4_ nanoparticles; secondly, the wide size distribution of Fe_3_O_4_ nanoparticles in the nanofibers, as shown in Figure 3. The size, aggregation and uniformity of particles play a very important role in the magnetic properties [35]. The saturation magnetization of 0.25 g/mL PVP/Fe_3_O_4_ membranes is larger than that of 0.2 g/mL PVP/Fe_3_O_4_ membranes. *M_s_* is proportional to the amount of Fe_3_O_4_ in the composite nanofibers [38]. The coercive forces of Fe_3_O_4_ nanoparticles and Fe_3_O_4_/PVP composite nanofibers are all 105 G at room temperature as shown in Figure 6b. The remanence of pure Fe_3_O_4_ particles, 0.25 g/mL and 0.2 g/mL PVP/Fe_3_O_4_ membranes are 10 emu/g and 7 emu/g, and 6 emu/g, respectively. It indicates that ferromagnetic responses are expected at room temperature. A PVP/Fe_3_O_4_ membrane at Fe_3_O_4_ concentration of 0.25 g/mL was needleless electrospun, as shown in Figure 6c. One end of the membrane was fastened onto the surface of a table, while the other end was free to move. The magnetic field responsive behavior of this PVP/Fe_3_O_4_ membrane with a N40 permanent magnet was observed in Figure 6c,d. As the magnet came closer to the membrane, a greater magnetic field gradient was induced to transfer larger magnetic force on membrane, causing the free end of the membrane to move towards the magnet. This demonstrated that the PVP/Fe_3_O_4_ membrane had a good magnetic response property.

## 3. Materials and Methods

### 3.1. Preparation of PVP/Fe_3_O_4_ Ferrofluid

Poly(vinyl pyrrolidone) (PVP, M_w_ = 1,300,000) was purchased from Aladdin Industrial Corporation, Shanghai, China. PVP was dissolved in the ethanol (99.5%, Guangzhou Chemical Reagent Factory, Guangzhou, China) at a concentration of 7% under sufficiently stirring for about 12 h at room temperature. Fe_3_O_4_ nanoparticles (99.5%; average diameter: 20 nm; saturation magnetization: 400 Gauss; Aladdin Industrial Corporation, Shanghai, China) were purchased as ferrite magnetic particles. Magnetite (Fe_3_O_4_) has been widely used in biomedical applications, such as magnetic resonance imaging (MRI), drug delivery, biosensors, magnetic separation, and medical diagnostics due to its innate biological compatibility [39]. The surface of the magnetic particles was modified by sodium oleate (98%, Shanghai Macklin Biochemical Co., Ltd., Shanghai, China).

Fe_3_O_4_ particles were dispersed in deionized water at 4% (*w*/*v*) by ultrasonic stirring at 70 °C. Subsequently, the surfactant sodium oleate was slowly added into the Fe_3_O_4_ /water solution at a ratio of 1:4 (*w*/*v*) and stirred for about 45 min. The modified Fe_3_O_4_ particles were precipitated using a strong NbFeB magnet and washed by ethanol solution several times to remove excessive sodium oleate. The modified Fe_3_O_4_ particles with sodium oleate were dried. The concentration of PVP/ethanol solution (7%) was prepared, because it was a proper concentration for the electrospinning of PVP nanofibers and good dispersion of Fe_3_O_4_ particles. The modified Fe_3_O_4_ particles were added to the PVP/ethanol solution at the concentrations of 0.2 g/mL and 0.25 g/mL in this experiment. Then the solution was stirred and dispersed by vortex and ultrasound. A relatively homogeneous polymeric PFF was prepared. The PFF was served as a raw solution for electrospinning of magnetic nanofibers.

### 3.2. Design of Needleless Electrospinning Apparatus

**Figure 7:**
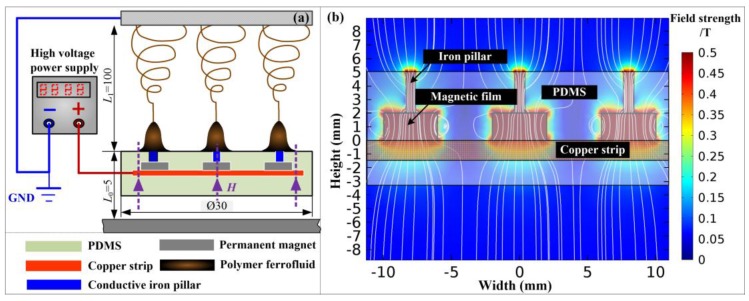
(**a**) Schematic image of needleless electrospinning apparatus, and (**b**) the magnetic field distribution of apparatus calculated by finite element analysis (FEA).

A needleless electrospinning apparatus for the preparation of magnetic nanofibers was self-developed, as shown in Figure 7a. The apparatus was composed of the magnetic field generator module and high electric field generator module. A 3-mm-height, 0.5-mm-diameter pure iron pillar was vertically and magnetically attracted by a 1.1-mm-thickness, 1.9-mm-diameter grade-N40 small permanent magnet. The small permanent magnets were connected with a copper strip. The pillars, small permanent magnets, and copper strip were totally embedded in a 30-mm-diameter solidified polydimethylsiloxane (PDMS) block. Only the top surface of the pillar end was exposed to air and directly contacted the PFF. The high electric field was generated by a high-voltage power supply (DW-P303-1ACF0, Tianjing Dongwen High Voltage Power Supply Plant, Tianjing, China) and directly applied on the PFF through the iron pillars. The collector was placed 10 cm away from the top surface of PDMS block and connected to the ground electrode of the high-voltage power supply. 

A 6-mm-thickness, 40-mm-diameter grade-N40 large permanent magnet was placed below the PDMS block with a distance of approximately 5 mm. This arrangement created a magnetic field directed normally to the top surface of PDMS block with a uniform flux intensity (*H*) of 75 mT. The pure iron pillar was further magnetized by the small permanent magnets. The residual flux intensity (*B_r_*) and relative magnetic permeability (*μ_r_*) of small magnets were 1.24 T and 1.05, respectively. The relative magnetic permeability of the iron pillar was 4000. The PFF was magnetized to form a cone droplet. The magnetic field distribution of the apparatus was numerically calculated with COMSOL Multiphysics 5.0 softwarefigure, as shown in Figure 7b. The simulation model was built based on the physical size of apparatus. The equivalent magnetic charge method was employed to calculate the distribution of the magnetic field.

### 3.3. Fabrication of Magnetic Nanofibers

The needleless electrospinning procedures for the preparation of magnetic nanofibers were as follows: (1) about 2 µL PFF was poured on a PDMS block with a pipette at each top end of the pillar. (2) The PFF was self-assembled into a droplet cone under the magnetic field. (3) A high-voltage power supply was adjusted at 28 kV and a high electric field was applied to the PFF cone-shape-droplets. The needleless electrospinning occurred and the nanofibers were collected. (4) This needleless electrospinning process was observed by a high-speed camera with a 10× lens (Fastec TS3-Model 100-S/L, Fastec Imaging Corporation, San Diego, CA, USA). (5) The nanofibers were vacuum dried for 24 h and stored in a vacuum-sealed desiccator to prevent the absorption of moisture.

The conventional needle electrospinning of PFF was also employed to prepare magnetic nanofibers. The applied voltage was 12 kV, the tip-to-collector distance was 10 cm and the mass flow rate was 0.8 mL/h. Both the needleless and needle electrospinning were performed at room temperature (25 °C) under a relative humidity of 40–45%.

### 3.4. Characterization Methods

The prepared magnetic nanofibers were characterized and analyzed by SEM, TEM, TGA, FT-IR, and VSM. The morphology of the resulting nanofibers was observed using a field emission scanning electron microscope (FESEM, JSM-6330F, JEOL Ltd., Tokyo, Japan). The internal morphology of composite nanofibers was studied using a transmission electron microscopy (TEM, Tecnai 10 Philips, Amsterdam, The Netherlands). The diameter of nanofibers was measured by Image-J image analysis software (National Institutes of Health, Bethesda, MD, USA) with about 100 randomly-selected nanofibers from each SEM image. Thermogravimetric analysis of nanofibers was carried out on a TGA instrument (TGA/SDTA85e, Mettler Toledo, Zurich, Switzerland) with a heating rate of 10 °C/min from 25 °C to 700 °C under a nitrogen atmosphere of 50 mL/h. The functional groups of nanofibers were studied by FT-IR (TENSOR27 Bruker, Karlsruhe, Germany). The magnetization versus the applied magnetic field curves (M-H curves) of magnetic nanofibers and Fe_3_O_4_ particles were evaluated using a vibrating sample magnetometer (VSM, Lake Shore 7410, Westerville, OH, USA) at room temperature.

## 4. Conclusions

We presented a novel needleless electrospinning method for the preparation of magnetic nanofibers from the self-assembling ferrofluid cone array under simultaneous magnetic and electric fields. The PVP/Fe_3_O_4_ ferrofluid was prepared for the needleless electrospinning. When the PFF droplet is subject to a magnetic field, the PFF shows a static Rosensweig instability with a conical protrusion on the free surface. When the surface is further stressed by an electric field, the tip of the protrusion emits an electrospinning jet. Both the electric and magnetic effects act in concert to draw the meniscus, ultimately causing the PFF jetting and electrospinning. The needleless electrospinning shows a similar process phenomenon in comparison with the conventional electrospinning. The needleless electrospinning jet becomes finer, and is finally deposited on the collector as the magnetic nanofibers. The nanofibers prepared by the needleless electrospinning show similar morphology in comparison with conventional electrospinning. The Fe_3_O_4_ particles distribute and aggregate both inside and on the surface of the nanofibers. The magnetic nanofibers consist of Fe_3_O_4_ particles and PVP polymer according to the analysis of TGA and FT-IR experiments. The nanofiber membrane also exhibits good ferromagnetic and magnetic field responsive properties. The magnetic nanofibers have potential in biomedical applications.
